# A Novel Infant’s Nursery

**Published:** 1866-12

**Authors:** 


A Novel Infant's Nursery.—An old stable with 100 infants in the horse-troughs and hay-cribs is rather a novelty, but it is seen in the locality of Union street, Borough-road, London. The work has been undertaken by the Rev. George Addington, who has secured an old stable to form a nursery, and has fitted it up for taking care of the young children of women who are obliged to go out to char or work away from home.—[Med. Press and Circular, October 31, 1866.

